# Augmentation therapy for alpha-1 antitrypsin deficiency: towards a personalised approach

**DOI:** 10.1186/1750-1172-8-149

**Published:** 2013-09-24

**Authors:** Robert A Stockley, Marc Miravitlles, Claus Vogelmeier

**Affiliations:** 1 Lung Investigation Unit, Queen Elizabeth Hospital Birmingham, Mindelsohn way, Edgbaston, Birmingham B15 2WB, UK; 2 Pneumology Department, Hospital Universitari Vall d’Hebron, Barcelona, Spain; 3 Universitätsklinikum Gieβen und Marburg, Standort Marburg, Baldingerstraβe, D-35043, Marburg, Germany

**Keywords:** Alpha-1 antitrypsin deficiency, COPD, Risk factors, Augmentation therapy

## Abstract

**Background:**

Intravenous augmentation therapy is the only specific treatment available for emphysema associated with alpha-1 antitrypsin deficiency. Despite large observational studies and limited interventional studies there remains controversy about the efficacy of this treatment due to the impracticality of conducting adequately powered studies to evaluate the rate of decline in lung function, due to the low prevalence and the slow progression of the disease. However, measurement of lung density by computed tomography is a more specific and sensitive marker of the evolution of emphysema and two small placebo-controlled clinical trials have provided evidence supporting a reduction in the rate of decline in lung density with augmentation therapy.

**The problem:**

Where augmentation therapy has become available there has been little consideration of a structured approach to therapy which is often introduced on the basis of functional impairment at diagnosis. Data from registries have shown a great variability in the evolution of lung disease according to patient acquisition and the presence of recognised risk factors. Avoidance of risk factors may, in many cases, stabilise the disease. Since augmentation therapy itself will at best preserve the presenting level of lung damage yet require intravenous administration for life with associated costs, identification of patients at risk of continued rapid or long term progression is essential to select those for whom this treatment can be most appropriate and hence generally more cost-effective. This represents a major reconsideration of the current practice in order to develop a consistent approach to management world wide.

**Purpose of this review:**

The current review assesses the evidence for efficacy of augmentation therapy and considers how the combination of age, physiological impairment, exacerbation history and rate of decline in spirometry and other measures of emphysema may be used to improve therapeutic decision making, until a reliable predictive biomarker of the evolution of lung impairment can be identified. In addition, individual pharmacokinetic studies may permit the selection of the best regimen of administration for those who need it.

**Summary:**

The rarity and variable characteristics of the disease imply the need for an individualised approach to therapy in specialised centres with sufficient experience to apply a systematic approach to monitoring and management.

## Introduction

Our understanding of the pathophysiology of COPD, but in particular emphysema was largely instigated by the observation that α_1_ antitrypsin deficiency (AATD) was associated with the early onset of basal panlobular emphysema [1]. Since AAT became characterised as an inhibitor of serine proteinases, data eventually showed that neutrophil elastase (NE) could produce emphysema like lesions in animal models [2]. Indeed subsequently this enzyme has been shown to produce many of the pathological features of COPD [3]. The logical conclusion from such studies was that augmentation of AAT in deficient subjects would restore the protection of the lung from NE and hence slow the aggressive form of emphysema seen in deficient subjects.

Because of the “rarity” of AATD it was deemed that classical clinical trials using spirometry as an outcome could not be undertaken [4] but the logical argument for efficacy, based on an understanding of the biochemistry prevailed. For these reasons augmentation therapy for AATD subjects became accepted and funded in many countries whilst others (unrealistically) called for conventional placebo controlled clinical trials to establish efficacy beyond doubt. However the high cost of such therapy means that efficacy is now being increasingly questioned even where therapy has been available and indeed some countries have withdrawn the use of augmentation whilst others continue to withhold therapy.

### So what is the data?

There is no doubt that augmentation therapy given intravenously increases the nadir antigenic AAT level to one that is consistent with the lower level for the heterozygote (MZ pheno/genotype) that carries no or, at least, very little risk to developing significant COPD [5], and above that for the SZ heterozygote where controversy still continues about whether such subjects are or are not at increased risk. Biochemical studies confirmed that at least some of the infused AAT remained active when retrieved from the lung by bronchoalveolar lavage [6] implying that it was also active in the lung tissues where the emphysema damage is thought to take place. A somewhat missed opportunity to verify the protective effect of augmentation therapy is the lack of definitive evidence that biomarkers of connective tissue degradation thought to be central to the development of emphysema, such as desmosines in plasma or more specific peptides derived from the microenviromental activity of neutrophil elastase, decline after initiation of intravenous augmentation therapy. New recent attempts to use these markers [7,8] could strengthen a personalised approach to treatment by ensuring markers of tissue damage are normal or become normal on therapy. However, it should be pointed out that biochemical efficacy based on AAT levels is not necessarily the same as clinical protection. The classical endpoint for clinical trials in COPD has long been the FEV_1_ and early calculations indicated that no study of augmentation could be powered for such an outcome due to the rarity of the disease [4].

**Figure 1:**
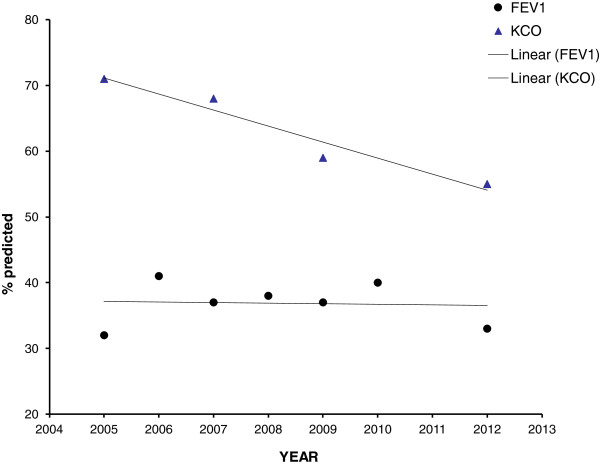
The decline in FEV1 and Kco expressed as a % predicted is shown over time for a 43 year old female from the UK registry who stopped smoking after diagnosis in 2005.

For this reason observational studies were undertaken and these indirect data were used to support efficacy. For instance, the NIH registry [9] suggested that individuals who received therapy for at least 6 months had not just a reduction in mortality but also a modulation of the FEV_1_ decline for those with baseline values in the range 35-60% predicted. However, these data were likely influenced, at least in part, by availability of healthcare provision and social aspects of healthcare delivery in the USA. Nevertheless, other studies provided similar results by observing a greater decline in FEV_1_ in countries where augmentation was not available [10] and a reduction in decline after therapy was instigated [11]. Furthermore the former observation was also supported by a recent meta-analysis providing indirect evidence that spirometric decline is less where augmentation is available [12]. Nevertheless it is recognised that these observations, though supportive, cannot replace formal clinical trials. Importantly the NIH study, where used to support augmentation, has been interpreted as suggesting no benefit outside the FEV_1_ limits of 35-60% predicted and in some countries augmentation is stopped below this lower limit and not usually started above these limits. However recent data has indicated that using other more specific and sensitive measures of emphysema, such as the alveolar gas transfer and/or the decrease in lung density, indicate that progression of lung disease occurs both above and below these FEV_1_ limits [13,14], even when FEV_1_ remains stable as indicated in data summarised in Figure 1 for an individual patient from the UK National Registry.

A recent Cochrane review took the pragmatic approach of analysing the efficacy of augmentation therapy based on the results of decline in FEV1 of the only 2 small placebo controlled trials available [15,16] and concluded that there was no convincing data to support the efficacy of augmentation therapy [17]. However, both studies were just short of conventional statistical significance in favour of the efficacy of augmentation therapy using lung densitometry as the outcome. Combining the 2 studies and excluding all individuals who participated in the first (less robust study) from the second (more robust study) was however highly suggestive of efficacy in reducing the rate of decline in lung density [18], which is validated and has become accepted as the most specific and sensitive measurement of the progression of emphysema [13,19,20]. In addition even the second study alone [16] was significant if analysis was confined to the lower zone where the panacinar emphysema of AATD predominates [21].

This disparity between a demonstrable effect on densitometry and a lack of effect on FEV1 is far from surprising. Not only were these studies based on assessing lung density, the most sensitive parameter to assess and monitor emphysema, but were not designed or powered to assess spirometric outcome. This raises a concern over the original NIH observation that did indicate a positive effect on FEV1 decline but only in a limited FEV1 range [9].

Recent understanding of the complexity of usual COPD, as well as of the lung disease associated with AATD has indicated the range of different pathological and clinical phenotypes [22]. Effective therapies can only be demonstrated easily if the generic COPD population is enriched for those with amplified evidence of the presence and progression of the proposed outcome measure. Since the FEV1 progresses most rapidly in the 35-60% predicted range [14] it would be the most sensitive range to detect a treatment effect with FEV1 as the outcome. In contrast, FEV1 decline is modest in severe disease, unlike the lung transfer coefficient for carbon monoxide (Kco) decline, which is greatest in severe disease [14]. Therefore, it is essential, especially with expensive therapy, to identify the patients particularly at risk and hence most likely to benefit from treatment by using outcomes specific to the disease process and to monitor efficacy where these are changing most. In the case of AATD, emphysema and not airflow obstruction is the primary pathophysiologic alteration, and it would be those with evidence of significant and progressing emphysema who should be selected for future efficacy trials. The assessment of emphysema should also be assessed by the most specific and sensitive test/s, in this case lung densitometry and alveolar gas transfer.

### Management paradigm

Patients with AATD can present with varying degrees of respiratory disease that is influenced by the awareness of the medical practitioner to the condition as well as the severity of symptoms by the time medical help is sought, together with a suggestive family history. Often there is a long lapse before AATD is diagnosed [23,24] and, especially in younger subjects, the symptoms may often be attributed to a more likely diagnosis of asthma. Patients may be identified as the index case presenting with symptoms or as non index, identified by family screening. The index group usually consists mainly of smokers especially if they are young and have the classical basal panlobular emphysema or if older and a non-smoker (ie no recognised risk factors) with fixed airflow obstruction. The non-index subjects identified through family screening usually have better lung function and includes both smokers and never smokers but still with a wide range of physiological impairment [25]. Interestingly these non-index patients may have complete discordance in their FEV_1_ with their index siblings but more concordance with gas transfer and lung densitometry [26]. This disparity provides further strong support for these latter measurements being more specific to AATD. A final smaller cohort can be identified due to perinatal jaundice, a recognised presenting feature of AATD [27] providing an opportunity for long term monitoring and earlier detection of deteriorating lung function.

This wide range of presenting age and features provides the managing physician with a challenge in determining the best care, monitoring and, importantly, deciding whether or when to introduce augmentation therapy. Such therapy cannot be expected to improve already damaged lungs and leads to the strategy of either preventing the development of lung pathology or stabilising that already present.

Currently augmentation is aimed at the latter approach of stabilising the established lung disease and thereby preventing future progression. It is recognised that at presentation with established disease, especially at a young age, the preceding period of the patients life must have been characterised by a decline in lung function that was in excess of the normal aging process. The first step in management however must be to stop smoking or endorse recent cessation, if that is the status. Usual management for COPD, such as bronchodilators, are prescribed or continued in order to maximise airflow physiology and/or reduce exacerbation frequency as in usual COPD. Indeed reversibility and exacerbations have been recognised as factors that influence spirometric [14,19] and gas transfer [19] decline in AATD and although few clinical trial data are available of the usual symptomatic and preventative therapies, they seem to be effective in AATD [28]. A summary of factors that have been associated with decline in FEV1 in patients with AATD is presented in Table 1.

**Table 1:** Factors influencing the natural course of emphysema in patients with AATD

**Intrinsic factors:**	
Bronchial hyperresponsiveness	[29]
Bronchodilator reversibility	[9,14,19,30]
Infections in childhood	[31]
Exacerbations	[14,19,32]
Pneumonia	[29,33]
Chronic bronchitis	[29,30,34]
Lower body mass index	[14,35]
**Extrinsic factors:**	
Smoking	[9,29,30]
Professional exposure to dusts and fumes	[36,37]
Air pollution	[38]

Where available, augmentation is usually prescribed especially if the FEV_1_ is in range of 35-60% predicted (as suggested by the observational NIH study), where spirometric benefit has best been demonstrated. However, despite the preceding decline in spirometry being obviously excessive prior to diagnosis, after the cessation of smoking and introduction of usual therapy for COPD there is no certainty that disease progression will continue, particularly in patients with no other recognised risk factors for progression (e.g. professional exposure to dusts and fumes, bronchial hyperresponsiveness, frequent exacerbations, etc.) [14,19,32,36-39]. Measurement of lung physiology is complex and single measures are subject to patient effort (even for simple spirometry) and day to day variability. For these reasons a period of monitoring for subjects with FEV1 between 80 and 60% of predicted at detection of the deficiency should be undertaken from diagnosis over at least 2–3 years assessing all aspects of physiological health status, not only FEV1, to determine stability or instability before the decision about augmentation therapy is taken. Nevertheless, it is recognised that such a delay may be less appropriate in some patients with more severe impairment (FEV1 and/or KCO < 60%, values at which most subjects report respiratory symptoms) as preservation of lung function becomes more critical at advanced stages. Decisions for augmentation treatment need to be made at this point on a risk/benefit basis, as the lung destruction in emphysema is irreversible and the next future option is transplantation or the continued increased morbidity, health care utilisation and death of unabated progression. Factors such as age, health status, activity and need and ability to continue current life style will all influence this decision making and, in some, further observation of decline after smoking cessation and optimisation of other therapies may still be possible or even essential. The development and validation of specific biomarkers that could predict future progression will become essential if such a period of observation is to be avoided.

Management of non-smokers and the more elderly patients becomes easier in decision making as the interaction with cigarette smoking (and hence the benefits of cessation) will not complicate assessment of the preceding natural history or will have indicated a much slower course [40,41]. Thus current age, morbidity and physiology are key factors that will provide information on overall rate of progression of lung disease since the attainment of maximal lung function in the teens. With this information an estimate of the likely subsequent rate of progression and future morbidity and hence any benefit of stabilisation with augmentation therapy can be made with more confidence. Although it is recognised that in never smoking non index cases, life expectancy is essentially normal [42,43] it is not necessarily without significant morbidity. As an example, a predictive model for FEV1 and the presence of severe COPD developed with data from 372 individuals with AATD phenotype PiZZ has identified age, sex, pack-years of smoking, bronchodilator responsiveness, chronic bronchitis symptoms and index case status as significantly associated factors. The model explained 50% of the variance in FEV1 and showed an excellent discrimination for severe COPD [30]. These findings suggest that the classical criteria for augmentation therapy based only on diagnosis of the deficiency and the presence of emphysema/reduced FEV1 without any consideration of risks of poor future evolution, must be improved.

The subjects identified in childhood through neonatal or family screening present a unique challenge. Currently there is no evidence to suggest all such subjects will develop COPD/emphysema. Indeed the data suggest that such a cohort has reasonably normal physiology in their 30s [44]. However normal physiology does not mean no problem as the normal range for lung physiology is wide and individual subjects can undergo significant physiological decline whilst lung function is still within the normal range. Indeed recent data suggest that changes within the normal range can be detected as early as in the mid to late 20s in such subjects even if never smokers [45]. Thus if augmentation therapy is to be used in a preventative strategy it would be appropriate to consider earlier intervention in such subjects before significant disease and morbidity occurs. For these reasons it seems appropriate to obtain a baseline assessment of lung health in the mid to late teens and then monitor any deterioration on a frequent basis (perhaps every 2–3 years) so that deterioration within the normal range can be determined early. Providing no risk factors can be implicated, summary statistics using 3–4 data points will provide data likely to predict future progression. The time at which augmentation is introduced will require a cost/benefit appraisal although an argument could be made to wait at least until the development of mild symptoms or physiological deterioration below the normal range. Whichever approach is used, data on previous rate of decline will provide some evidence of efficacy determined by observation of the subsequent rate of decline of lung function.

### Should frequent exacerbations influence decision making?

Exacerbations of COPD have become widely recognised as episodes that can lead to a decline in spirometry, impairment in health status and increased risk of death [46]. Exacerbations caused by bacteria are neutrophilic and although largely confined to the airways, are associated with easily detectable excessive (or increased) NE activity [47]. The inflammation and amount of detectable NE is even greater in subjects with AATD [48] suggesting that NE generated progression is more likely in such patients and indeed there is a similar effect of exacerbations on spirometric decline as seen in usual COPD; furthermore, exacerbations are also associated with a decline in the gas transfer of the lung for carbon monoxide over time in patients with AATD [19,32]. Early retrospective analysis suggested that augmentation therapy reduced the number of exacerbations [49] although the increase in health care contact due to the regular infusions could have influenced this result. In the EXACTLE trial exacerbation frequency was not reduced, although there was a reduction in severe episodes requiring hospitalisation [16]. This observation is consistent with the increased inflammation associated with exacerbations in AATD and the ability of augmentation to reduce lung inflammation [50] thereby reducing the clinical severity of the episodes.

**Figure 2:**
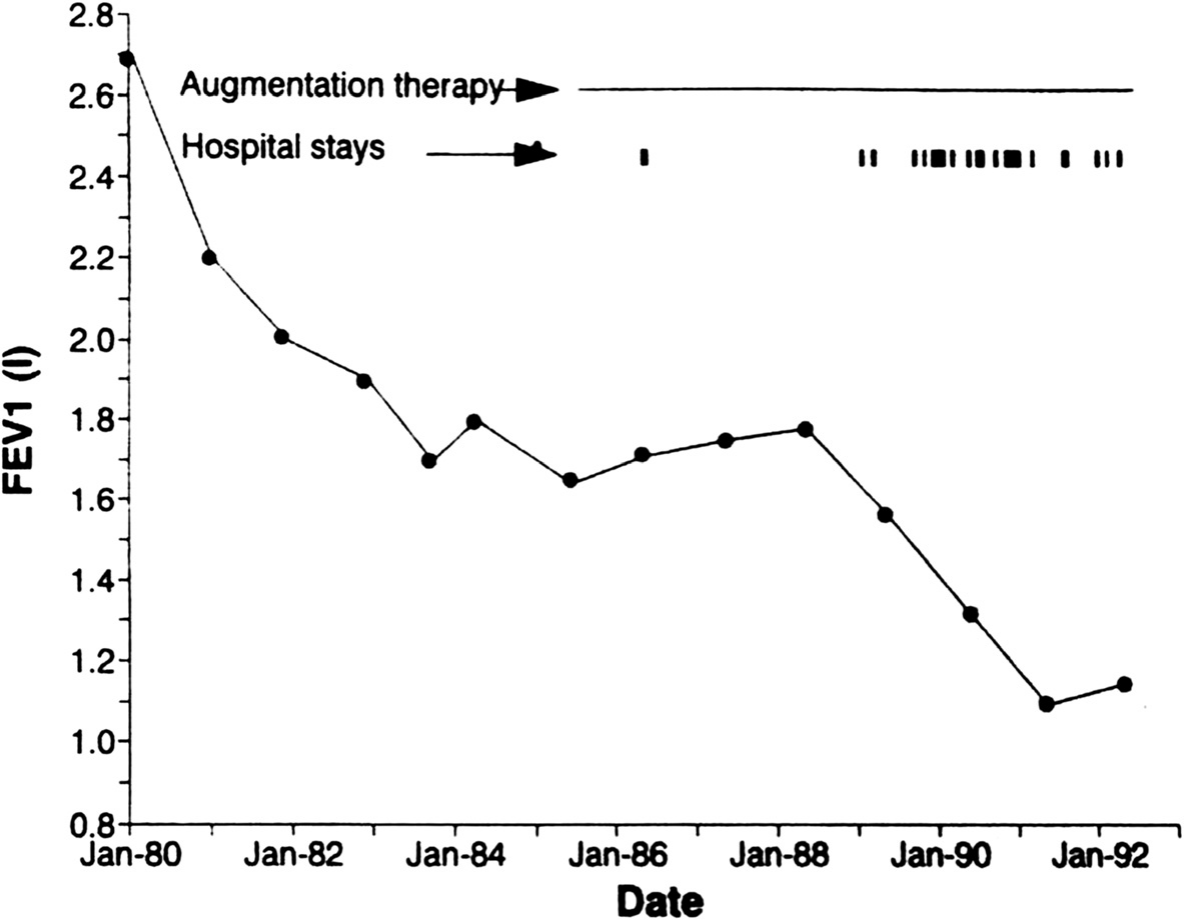
**The decline in FEV1 (l) for a 41 year-old male.** Footnote: Between 1980 and 1985 a severe drop in FEV1 from 2.7 to 1.7 liters was noted. Started with augmentation therapy in 1985, the lung function parameters stabilised over a period of approximately 3 years. The number of severe pulmonary infections requiring hospitalisation increased from 1989 and were accompanied by a rapid decrease in FEV1. Published from reference 46 with permission from S. Karger AG Basel.

The inflammatory burden associated with severe exacerbations may still accelerate the lung damage in patients with AATD, as illustrated in a published case study where, after a period of stabilisation of lung function with augmentation therapy, FEV1 decline accelerated after a series of admissions for exacerbations despite the continuation of the augmentation therapy [51] (Figure 2). This observation suggests that in some patients and/or in some situations the current dosage based on the stable state nadir plasma levels of heterozygotes or the route of administration may be insufficient to prevent lung deterioration [52] and perhaps bolus or inhaled therapy at the start of an exacerbation might prove effective [53]. Clearly further studies are needed to determine the validity of this approach.

Hospital admissions are expensive and associated with increased mortality in COPD [54,55]. Thus such AATD patients (despite usual COPD preventative therapy) may represent a more immediately cost effective group requiring augmentation therapy and frequency of admissions pre and post therapy can be monitored to indicate probable benefit or lack of benefit. However it may be that local administration of AAT by the inhaled route will prove most effective in frequent exacerbations as these are airway dominant episodes and not alveolar/interstitial processes, that may be beyond the reach of conventional nebulisers [56]. Results of the current ongoing trial in such patients may help resolve this issue [57].

Different series have demonstrated a prevalence of bronchiectasis of around 25% to 50% in patients with AATD [58,59]. The relationship between AATD and bronchiectasis is not fully elucidated. Although some studies suggest that patients with AATD are more at risk of developing bronchiectasis, large series of bronchiectasis patients have failed to demonstrate an increase of cases with AATD [60]. Moreover, the prevalence of bronchiectasis in patients with airflow obstruction is similar to that seen in usual COPD [61,62]. The presence of bronchiectasis is associated with increased risk of bronchial colonisation and hence airway inflammation, and more frequent and severe exacerbations in usual COPD [62,63]. Therefore, it is likely that AATD patients with infective complications such as frequent exacerbations, bronchiectasis, pneumonia or even chronic bronchitis may represent a subgroup with particular need for acute or long term augmentation therapy [29,33] as illustrated in the case 3 although perhaps again the airway route of administration may be more relevant.

### From the criteria for augmentation therapy to a personalised approach to treatment

Soon after the approval of augmentation therapy by the Food and Drug Administration, the scientific societies produced the first guidelines for augmentation, that included the classical criteria for treatment comprising, among others, the demonstration of airflow obstruction, a severe AAT deficiency (usually PI*ZZ or null genotypes), and abstinence of smoking [64]. Evidence has accumulated over several decades and summarised above to indicate that this approach may not be appropriate for all patients with the deficiency. The intravenous route of administration, the high cost of treatment and the differences in natural course and prognosis among patients, force us to be as precise as possible in recommending augmentation. This supports the idea of a personalised approach to treatment in reference centres with experts who can take into account all the characteristics of each individual with the deficiency and evaluate the future risks and eventually make the decision to initiate augmentation therapy based on a personalised evaluation of risks, benefits and costs.

**Figure 3:**
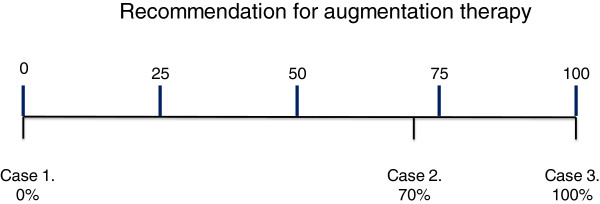
**Recommendation for augmentation therapy.** A new scale. Indications for augmentation therapy can vary from not to definitely indicated. Several factors will influence where the patient is placed on the scale including age, baseline lung function (FEV1 and/or Kco) and rate of decline expressed as a% predicted. A potential example is to divide the scale into quartiles with the lowest being age 60+ and/or lung function >80% predicted and/or decline < 0.1% predicted /year. The second quartile could be 50–60 years age and/or lung function 60-80% predicted and/or decline of 0.1-0.5% predicted /year. Third quartile 40–50 years age and/or lung function 40-60% predicted and/or decline of 0.5-1.0% predicted/year. Fourth quartile 30–40 years age and/or lung function 30-40% predicted and /or decline >1.0% predicted/year. This concept is summarised in Table 2. Footnote: Case 1. 75 years-old male, ex-smoker, or other non-index case with normal physiology. Indication for augmentation therapy 0% Case 2. 61 years-old male, exsmoker of 40 pack-years, index case. FEV1 = 58% predicted with KCO = 75% predicted. One ambulatory exacerbation the previous year. Previous spirometry one year ago FEV1 = 60% and 2 years ago = 62%. Indication for augmentation therapy 70%. Case 3. 42 years-old female, index case, exsmoker of 14 pack-years, FEV1 = 42% predicted and KCO = 32% predicted. Hospitalisation for exacerbation 5 months ago. Previous spirometry one year ago FEV1 = 51% predicted or patient with declining physiology despite smoking cessation and maximal usual therapy as in the patient in Figure 1 Indication for augmentation therapy 100%.

The adequacy of treatment can be represented by a continuous line from one end in which augmentation therapy would not be recommended, to the opposite end where all patients fulfilling those characteristics would be recommended to start augmentation therapy (summarised in proposed quartiles in Table 2). Each individual patient should be placed at a given point between these two opposite ends, which would indicate the strength of the recommendation for therapy (Figure 3). The threshold for recommendation of augmentation therapy could be established at a given point between both ends, always recommended or never recommended, based on the existing evidence of benefits of therapy, baseline presenting demographics, future known or projected prognosis and evaluation of costs [65]. At present decision making is largely instigated on a cross sectional basis. Ideally decision making should be with a clear understanding of the future predicted progression and that requires a period of observation once smoking cessation has been confirmed, all other known factors avoided and optimal COPD therapy instigated. Slow and fast decline based on expected changes with age can be used to place the subjects between the 2 ends of the treatment spectrum. Continued observation may enable the patient to be moved within the spectrum and this depends on a clearer understanding of the natural history both before and after diagnosis. Clearly urgent research is needed to clarify this approach in the few remaining cohorts where therapy has not been instigated or made available. It may be possible to develop a more objective scoring system based on a weighted score of the key factors of age, current severity and preceding rate of decline coupled with health economic data and examination of such an approach is urgently required to obtain international agreement and the development of firm guidelines.

**Table 2:** **Summary of factors influencing patient placement within Figure**3

**Parameter**	**Quartile 1**	**Quartile 2**	**Quartile 3**	**Quartile 4**
Age	60+	50-60	40-50	30-40
FEV1/Kco (% pred)	>80	60-80	40-60	30-40
FEV1/Kco decline (% pred/yr)	<0.1	0.1-0.5	0.5-1.0	>1.0

It remains important to state that this article is predominantly based on data and concepts applicable to the PiZZ and PiZnull genotypes of AATD. The PiSZ genotype has higher levels of serum AAT but lower than for the heterozygote MZ genotype. Few studies of the natural history of SZ patients or clinical trials data are available for such patients. Although some clinicians treat SZ patients in the same way as the more severely deficient ZZ and Znull subjects the SZ patients have a milder degree of impairment compared to matched PiZZ subjects and a more apical distribution of emphysema similar to usual COPD [66]. There is little clinical or theoretical data to support augmentation of SZ patients at present and further studies are urgently indicated.

Once therapy is indicated, the next step should be the adjustment of the right dose and interval of administration for each individual patient. Pharmacokinetic models will help individualise the regimen in order to provide adequate trough serum concentrations with the lowest cost [67]. However it should be noted that such a decision may need to take into account both the antigenic and importantly the functional quantity of AAT as the latter is the most relevant for lung protection.

## Competing interest

RAS has lectured and acted on the advisory boards of CSL Behring, Grifols, kamada and Baxter. He is also in receipt of an unrestricted research grant from Grifols. MM has received honoraria for participating in advisory boards and lecturing from Talecris-Grifols. He has acted as consultant for Kamada and CLS Behring. CV has received honoraria for participating in advisory boards and lecturing from Talecris-Grifols. MM has received honoraria for participating in advisory boards and lecturing from Talecris-Grifols. He has acted as consultant for Kamada and CLS Behring. CV has received honoraria for participating in advisory boards and lecturing from Talecris-Grifols.

## Authors’ contribution

RAS conceived of the idea and mainly wrote the manuscript and with MM developed the outline of the document. CV provided important input towards the final document as well as suggestions towards the development of the treatment algorithm. All authors read and approved the final manuscript.
